# Perioperative hormone level changes and their clinical implications in patients with pituitary adenoma: a retrospective study of 428 cases at a single center

**DOI:** 10.3389/fendo.2023.1286020

**Published:** 2023-10-30

**Authors:** Min Zhao, Kai Li, Hongchuan Niu, Yuanli Zhao, Changyu Lu

**Affiliations:** Department of Neurosurgery, Peking University International Hospital, Beijing, China

**Keywords:** pituitary adenoma, microscopic surgery, endocrine dysfunction, growth hormone, prolactin, postoperative endocrine deficiency

## Abstract

**Objective:**

This study employs case data analysis to elucidate alterations in hormone levels pre and post-surgery among patients with pituitary adenoma. Moreover, it investigates the influence of various associated factors on endocrine function.

**Methods:**

A retrospective analysis was conducted on clinical data from 428 patients who underwent surgical treatment within a short period at a single center. Statistical methods were employed to examine detailed hormone level fluctuations before and after surgery in patients with pituitary adenoma, along with their interrelations with different factors.

**Results:**

Between January 2016 and October 2022, a total of 428 consecutive patients with pituitary adenoma underwent surgical treatment. Of these, 232 were males and 196 were females, with an average age of 45.91 years (range: 16-84, standard deviation: 12.18). Univariate analysis indicated that females exhibited a higher susceptibility to preoperative endocrine dysfunction (p < 0.05). Conversely, males, patients with larger tumor volumes, and older patients were more prone to preoperative pituitary insufficiency. Postoperatively, the most substantial remission rate occurred in prolactin (77.4%), followed by growth hormone (GH) (71.8%) and cortisol (4/6). The highest recovery rate in functions was observed in growth hormone secretion function (80%), followed by pituitary-adrenal axis hormone secretion function (56.3%) and pituitary-thyroid axis hormone secretion function (47.5%). The most noteworthy incidence of newly developed postoperative endocrine deficiencies was found in the pituitary-adrenal axis (31.8%), while the occurrence rates of deficiencies in other axes were relatively low. The elevated postoperative remission rate of growth hormone correlated with a higher surgical resection rate and lower preoperative growth hormone levels. Additionally, lower preoperative prolactin levels corresponded to a higher remission rate of prolactin postoperatively. Furthermore, the restoration of postoperative thyroid hormone secretion function was associated with higher preoperative free thyroxine levels. Reduced postoperative cortisol secretion function was linked to multiple surgeries and an extended interval between hormone retesting and surgery.

**Conclusion:**

Surgical intervention effectively ameliorates endocrine disorders in pituitary adenoma patients, thereby mitigating symptoms and enhancing their quality of life. Preoperative management of growth hormone and prolactin levels facilitates an increased remission rate of these hormones post pituitary adenoma surgery. Patients displaying preoperative thyroid hormone secretion dysfunction should be considered for active supplementation therapy. Whenever feasible, complete tumor resection is recommended. For patients undergoing reoperation or multiple surgeries, vigilant postoperative cortisol monitoring and supplementation should be thoughtfully administered.

## 1 Introduction

Pituitary adenomas rank among the three most prevalent intracranial tumors. Predominantly benign, they account for approximately 12% of all intracranial neoplasms. Most are non-functional adenomas, primarily causing symptoms through mass effect and compression. This is evident in presentations such as headaches, vomiting, visual field deficits, and diminished vision. A minority are functional adenomas, characterized by symptoms arising from endocrine hormonal dysregulation. Depending on the specific hormonal disruption, they may manifest various symptoms including amenorrhea, galactorrhea, polydipsia, polyuria, central obesity, acromegaly, and gigantism etc. These symptoms have a substantial impact on the patient’s quality of life. Therefore, following a confirmed diagnosis, the majority of cases necessitate proactive interventions including medication, surgical procedures, gamma knife therapy, and other pertinent treatments.

Surgery stands as the foremost and pivotal therapeutic approach for pituitary adenoma (PA) so far, offering both relief from tumor compression and enhancement of endocrine function in many hormone-secreting tumor cases. A multitude of investigations have demonstrated that substantial proportions of elevated hormone levels can attain complete remission in instances of elevated serum hormone levels. The remission rates for distinct adenomas, namely prolactinomas, growth hormone-secreting adenomas, adrenocorticotropic hormone-secreting adenomas, gonadotropin-secreting adenomas, and thyroid-stimulating hormone-secreting adenomas, fall within the ranges of 46%-87%, 35%-80%, 71%-100%, 71%, and 100%, respectively. Even in situations where complete remission remains elusive, a considerable number of patients commonly experience partial enhancement in endocrine function (1–17). Nevertheless, surgical interventions can also engender postoperative pituitary dysfunction, occasionally necessitating hormone replacement therapy. This phenomenon can exert unfavorable effects on patients’ financial circumstances, daily routines, and even psychological well-being.

Studies focusing on cases exhibiting preoperative pituitary function within the normal range followed by subsequent postoperative dysfunction are notably limited (n < 50), and their duration extends significantly (12 years), (2) potentially introducing systematic errors related to measurement devices, diagnostic standards, and therapeutic approaches. Consequently, this approach might not accurately capture the factors influencing postoperative cortisol variations within the current context of continually progressing diagnostic and therapeutic standards.

In this investigation, we undertook a retrospective analysis of 428 cases involving surgically treated pituitary adenomas at a singular medical center spanning an 80-month period. Our primary objective was to delve into the alterations in hormone levels prior to and subsequent to surgery among patients with pituitary adenoma. Furthermore, we sought to elucidate potential factors that could exert influence, aiming to provide valuable clinical insights.

## 2 Cases and methods

### 2.1 Inclusion criteria

Patients with pathologically confirmed pituitary adenomas who underwent surgical treatment at Peking University International Hospital between January 2016 and October 2022 were consecutively enrolled. Basic demographic information, preoperative and postoperative hormone test results, clinical data before surgery, tumor size, surgical specifics, postoperative pathological classification, and clinical data were extracted from electronic medical records. Cases without hormone level test results pre-operation or within one week after surgery were excluded. Cases with missing imaging data for post-operative review were excluded. A statistical analysis was conducted to assess changes in serum hormone levels before and after surgery, along with their influencing factors.

### 2.2 Endocrine assessment

Preoperative and postoperative endocrine evaluations were performed in the hospital’s laboratory. Serums were collected before rising in the morning, immunoassays were utilized to measure hormones in the serums related to the pituitary-adrenal axis, pituitary-thyroid axis, pituitary-gonadal axis, serum prolactin, and growth hormone.

The evaluation of the pituitary-adrenal axis was based on serum cortisol concentration (normal range: 6.24-18.00 μg/dL in the morning, 2.69-10.40 μg/dL in the afternoon). Cortisol deficiency was defined as a serum cortisol concentration <6.24 μg/dL, while cortisol excess was defined as >18.0 μg/dL.

Evaluation of the pituitary-thyroid axis involved measuring serum free T4 (FT4) concentration (normal range: 12.00-22.00 pmol/L) and thyroid-stimulating hormone (TSH) concentration (normal range: 0.27-4.20 μIU/mL). Thyroid dysfunction was characterized by FT4 <12.00 pmol/L, regardless of TSH concentration (18).

Assessment of the pituitary-gonadal axis considered gender, age, serum luteinizing hormone (LH), follicle-stimulating hormone (FSH), estradiol, and testosterone concentrations. Dysfunction of this axis was determined based on hormone levels and age.

Prolactin secretion evaluation involved measuring serum prolactin (PRL) concentration (normal range: 3.46-19.4 ng/mL in males, 5.18-26.53 ng/mL in females). Hyperprolactinemia or hypoprolactinemia was defined by elevated or reduced PRL levels, respectively.

Evaluation of growth hormone secretion involved measuring serum growth hormone (GH) concentration (normal range: 0-2.47 ng/mL in males, 0.126-9.88 ng/mL in females) and insulin-like growth factor-1 (IGF-1) serum concentration (normal range: 94.4-223 ng/mL in males, 56.3-170 ng/mL in females). Elevated GH was defined by levels above the normal range (18).

Assessment of reduced antidiuretic hormone (ADH) secretion required a urine volume ≥40 mL/kg/day and urine osmolality <300 mOsm/kg. Due to a lack of relevant quantitative data, further discussion on ADH secretion reduction was omitted.

Pituitary deficiency was defined by the presence of one of the following conditions: global pituitary deficiency (deficiency in all axes), single-axis deficiency (deficiency in any single axis), two-axis deficiency (deficiency in two axes), or three-axis deficiency (deficiency in three axes).

### 2.3 Postoperation hormone changes

All patients underwent postoperative serum hormone level reassessment within one week, with blood samples collected between 6:00 and 8:00. Methylprednisolone sodium succinate (methylprednisolone) intravenous infusions of 0-80 mg were administered on the surgery day, followed by additional doses for the subsequent three days. Given methylprednisolone’s short half-life (1.9-5.4 hours), its impact on morning serum cortisol measurements was deemed negligible (19–21). Postoperative hormone changes were classified as follows: a) Recovery: Normalization of previously deficient axes postoperatively. b) Remission: Normalization or reduction of previously elevated axes postoperatively (22). c) Deficiency: Transition of previously normal axes to deficiency postoperatively.

### 2.4 Imaging evaluation

**Figure 1:**
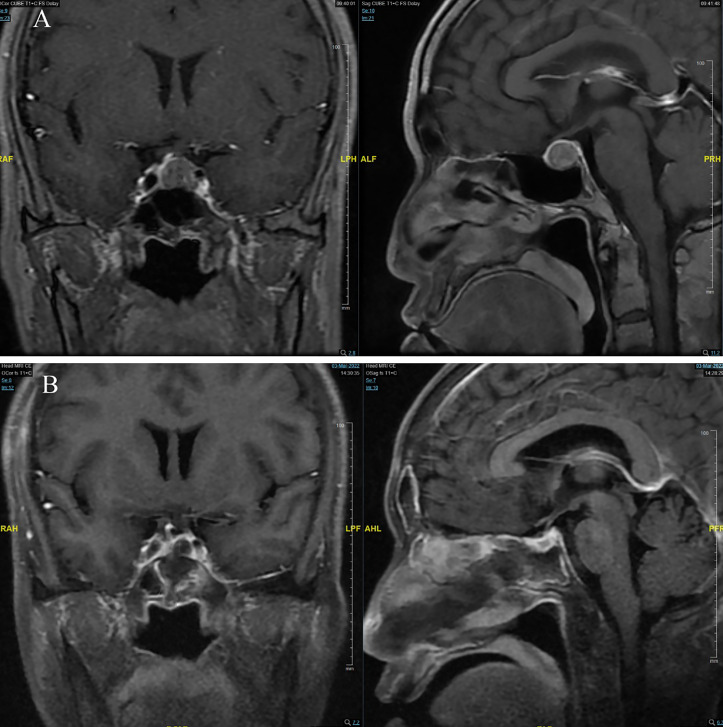
**(A)** pre-operation; **(B)** post-operation.

**Figure 2:**
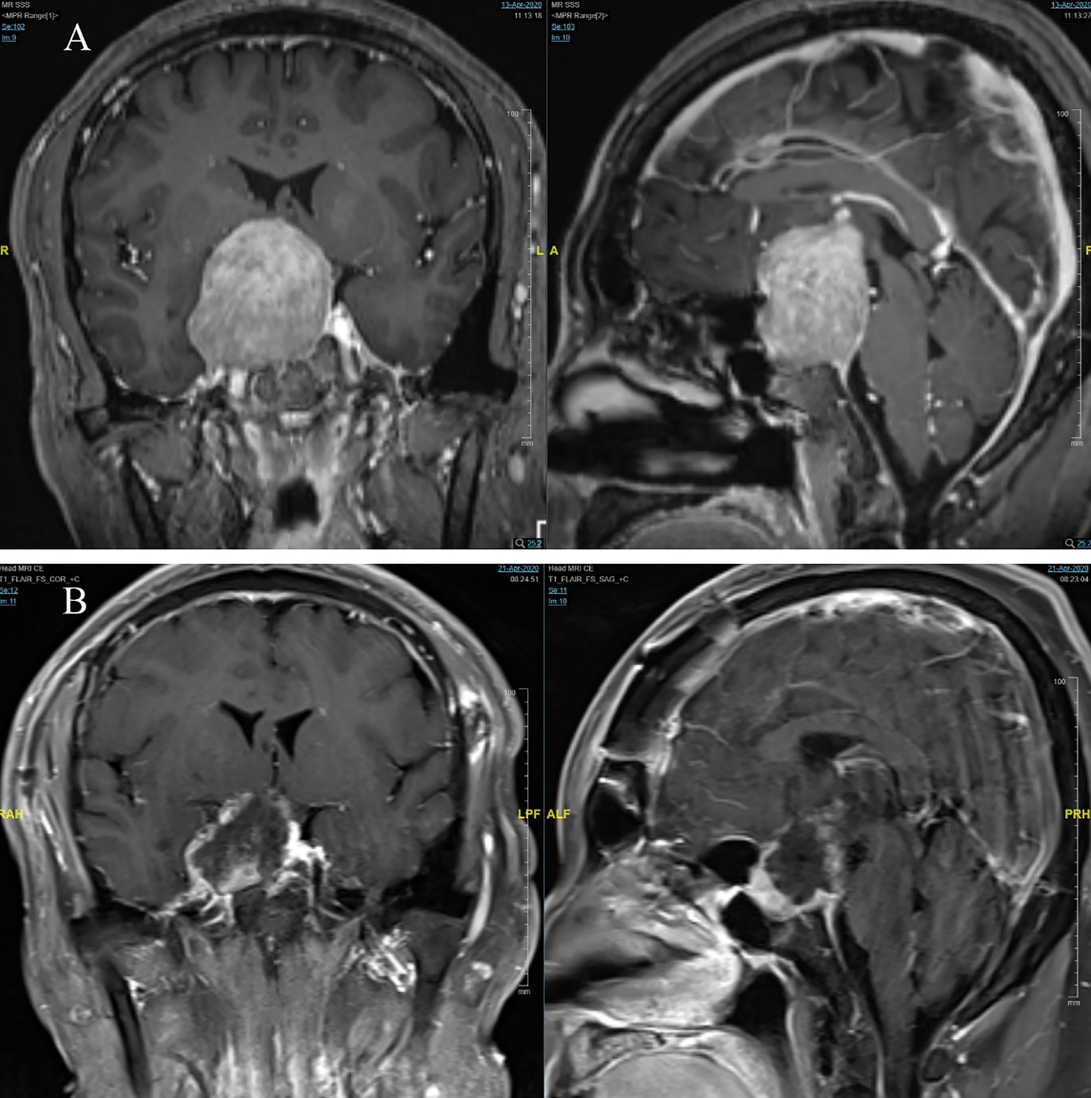
**(A)** pre-operation; **(B)** post-operation.

All patients underwent 3.0T cranial magnetic resonance imaging (MRI) scans (5mm slice thickness) before and after surgery at our institution. For certain microadenoma cases, dynamic enhanced scans of the sellar region were conducted before surgery to ascertain tumor size and location. Evaluation of tumor characteristics, encompassing size and hemorrhage occurrence, was performed by at least one neuroimaging specialist and multiple neurosurgeons. Tumor size was categorized as microadenoma (maximum diameter d ≤ 10mm), macroadenoma (10mm<d ≤ 30mm), and giant adenoma (d>30mm). Postoperative imaging assessments determined tumor resection extent, classified as gross total resection (**Figures 1A, B**), near-total resection (resection≧90%, **Figures 2A, B**), or partial resection(resection<90%).

### 2.5 Tumor resection surgery

Tumor resection surgeries were conducted by experienced neurosurgeons with over a decade of expertise at our center. The choice of surgical approach—transcranial (**Figure 2A**) or endonasal transsphenoidal (**Figure 1A**)—was predicated on tumor size, location, and other considerations. Surgeries were guided by microscopy or neuroendoscopy. The primary objective was maximal tumor resection, while mindful of scenarios where tumor proximity to vital nerves, vessels, or normal pituitary tissue necessitated restraint. Instances of intraoperative sellar floor disruption or cerebrospinal fluid (CSF) leakage during endonasal transsphenoidal approaches entailed repair utilizing autologous fat and muscle with bioadhesive. In minor leakage cases, gelatin sponge and bioadhesive were employed. Data compilation encompassed surgical approach details, procedure duration, occurrences of intraoperative CSF leakage, need for blood transfusion, and postoperative localized hematoma events.

### 2.6 Pathology and immunohistochemistry

Following surgery, specimens underwent hematoxylin and eosin (HE) staining and immunohistochemical examination in our hospital’s pathology department. The ultimate pathological diagnosis was pituitary adenoma. Based on immunohistochemistry positive results, specimens were further categorized as non-functioning type (completely negative), growth hormone-secreting type (GH-positive), prolactin-secreting type (PRL-positive), adrenocorticotropic hormone-secreting type (ACTH-positive), thyroid-stimulating hormone-secreting type (TSH-positive), follicle-stimulating hormone-secreting type (FSH-positive), luteinizing hormone-secreting type (LH-positive), or mixed type (positive for two or more hormones).

### 2.7 Statistical analysis

Statistical analysis employed IBM SPSS Statistics version 19.0. Descriptive analysis encompassed patient characteristics with preoperative hypofunction, normal function, or hyperfunction of each axis. The relationship between these characteristics and clinical factors was examined through univariate analysis. Similarly, descriptive analysis was applied to patients with postoperative recovery, improvement, or deterioration of endocrine function in each axis. Independent sample univariate analysis was conducted to identify potential factors influencing postoperative changes. Numerical variables (e.g., age, maximum tumor diameter, surgical duration) were subjected to t-tests, analysis of variance (if normally distributed), or Mann-Whitney U tests (if not normally distributed). Categorical variables (e.g., gender, age stratification, tumor pathology, surgical approach, surgical duration stratification) were analyzed using chi-square tests or Fisher’s exact probability method. Finally, logistic regression analysis was performed on variables with P<0.05 in univariate analysis to ascertain potential influencing factors for postoperative recovery, improvement, or deterioration of endocrine function in pituitary adenoma patients.

## 3 Results

### 3.1 Basic patient characteristics

Between January 2016 and October 2022, a total of 428 patients with pituitary adenomas underwent surgical treatment, with 54.2% being male and 45.8% female. The average age was 45.91 years (range: 16-84, standard deviation 12.18). The summarized general characteristics can be found in **Table 1**. Among these patients, 253 presented with visual impairments, 136 reported headaches, 75 exhibited acromegaly, 9 displayed signs of Cushing’s syndrome, 60 had undergone prior surgeries (52 cases once, 8 cases twice), 40 had a history of diabetes, and 94 had hypertension. No patients had received previous radiation therapy. Effective cases for each endocrine axis (including growth hormone and prolactin) are presented in **Table 2**, excluding cases without preoperative or within one-week postoperative hormone test results. Among these, 412 cases involved the pituitary-adrenal axis, 401 cases the pituitary-thyroid axis, 341 cases the pituitary-gonadal axis, 415 cases had assessable growth hormone secretion, and 417 cases had assessable prolactin secretion. The male-to-female ratio was nearly balanced, with a slight preponderance of males.

**Table 1:** Basic characteristics of 428 patients undergoing surgical treatment.

Characteristics (n=428)	Number of Cases (%)
Gender	
Male	232 (54.2)
Female	196 (45.8)
Visual Impairment	253 (59.1)
Headache	136 (31.8)
Menstrual Disorders/Galactorrhea/Sexual Dysfunction	155 (36.2)
Cushing’s Syndrome	9 (2.1)
Acromegaly	75 (17.5)
Comorbid Hypertension	94 (22.0)
Comorbid Diabetes	40 (9.3)
Times of Previous Surgery	
1 surgery	52 (12.1)
2 surgeries	8 (1.9)
Tumor Size	
Microadenoma	23 (5.4)
Macroadenoma	256 (59.8)
Giant Adenoma	149 (34.8)
Tumor Pathological	
Non-functioning	139 (32.5)
Mixed Type	135 (31.5)
GH-Secreting Type	16 (3.7)
PRL-Secreting Type	33 (7.7)
ACTH-Secreting Type	40 (9.3)
TSH-Secreting Type	2 (0.5)
FSH-Secreting Type	53 (12.4)
LH-Secreting Type	10 (2.3)
Tumor Apoplexy	39 (9.1)
Pathological Immunohistochemical	
PRL Positive	141
All Negative	139
GH Positive	112
FSH Positive	84
ACTH Positive	74
TSH Positive	47
LH Positive	23

**Table 2:** Effective cases of various axes in 428 cases of surgical treatment.

Endocrine Axis	Number of Cases (%)
Pituitary-Adrenal Axis	412
Male	224 (54.4)
Female	188 (45.6)
Pituitary-Thyroid Axis	401
Male	218 (54.4)
Female	183 (45.6)
Pituitary-Gonadal Axis	341
Male	186 (54.5)
Female	155 (45.5)
Growth Hormone	415
Male	226 (54.5)
Female	189 (45.5)
Prolactin	417
Male	228 (54.7)
Female	189 (45.3)

The mean tumor diameter among the 428 cases was 27.83 mm (range: 3-80, standard deviation 11.46). Of these, 23 cases (5.4%) were microadenomas, 256 cases (59.8%) were macroadenomas, and 149 cases (34.8%) were giant adenomas. Moreover, 39 cases (9.1%) had experienced tumor-related hemorrhages before surgery. Postoperative pathology and immunohistochemistry classifications revealed the following distribution: non-functioning type (139 cases, 32.5%) was the most common, followed closely by the mixed type (135 cases, 31.5%). The remaining cases were categorized as follows: FSH type (12.4%), adrenocorticotropic hormone (ACTH) type (9.3%), prolactin (PRL) type (7.7%), growth hormone (GH) type (3.7%), luteinizing hormone (LH) type (2.3%), and thyroid-stimulating hormone (TSH) type (0.5%). Among the cases classified as mixed type, the immunohistochemistry-positive results for different hormone types were as follows: PRL positive in 141 cases, completely negative in 139 cases, GH positive in 112 cases, FSH positive in 84 cases, ACTH positive in 74 cases, TSH positive in 47 cases, and LH positive in 23 cases.

### 3.2 Preoperative pituitary endocrine function

Prior to surgery, a total of 11 cases (2.5%) exhibited combined low function of the pituitary-adrenal, pituitary-thyroid, and pituitary-gonadal axes. Among these, two cases underwent serum IGF-1 level testing, both yielding results below the normal range. Among the 11 patients, one case displayed elevated serum growth hormone levels (16.4 ng/ml, male) with no available IGF-1 test results. Excluding this growth hormone elevation, 10 cases (2.3%) had preoperative growth hormone secretion deficiency.

As depicted in **Table 3**, normal pituitary function and serum prolactin levels were present in 101 cases (23.6%) before surgery. The primary categories were non-functioning type (39.6%), mixed type (25.7%), ACTH type (13.9%), and FSH type (11.9%). A total of 144 cases (33.6%) exhibited elevated hormone levels in at least one axis, with 137 cases (32.0%) displaying elevated prolactin levels. Preoperative deficiency in at least one pituitary axis was observed in 126 cases (29.6%). Of these, 90 cases (21.0%) had single-axis deficiency, with the pituitary-gonadal axis being most affected (53.3%). Additionally, 25 cases (5.8%) exhibited deficiency in two axes, most commonly the pituitary-adrenal and pituitary-thyroid axes (56%). One case demonstrated deficiency in three axes (accompanied by elevated serum growth hormone), while 10 cases (2.3%) had panhypopituitarism. Sixteen cases (3.7%) exhibited hypoprolactinemia.

**Table 3:** Preoperative hormone status of 428 patients undergoing surgical treatment.

Hormone Distribution (n=428)	Number of Cases (%)
Pituitary-Adrenal Axis	
Cortisol Deficiency	71 (16.6)
Hypercortisolism	6 (1.4)
Cortisol Insufficiency	5 (1.2)
Pituitary-Thyroid Axis	
Hypothyroidism	40 (9.3)
Hyperthyroidism	5 (1.2)
Thyroid Hormone Insufficiency	14 (3.3)
Pituitary-Gonadal Axis	
Hypogonadism	70 (16.4)
Elevated FSH/LH	67 (15.7)
FSH/LH Insufficiency	24 (5.6)
Growth Hormone	
Elevated Growth Hormone	71 (16.6)
Reduced Growth Hormone	10 (2.3)
Growth Hormone Insufficiency	4 (0.9)
Prolactin	
Hyperprolactinemia	137 (32.0)
Hypoprolactinemia	16 (3.7)
Prolactin Insufficiency	5 (1.2)
Normal Preoperative Endocrine Hormones	101 (23.6)
Non-functioning	40 (39.6)
Mixed Type	26 (25.7)
ACTH-Secreting Type	14 (13.9)
FSH-Secreting Type	12 (11.9)
Other	9
Elevation of Any Axis Hormone^a^	144 (33.6)
Hypofunction of Pituitary Axes ^b^	126 (29.6)
Panhypopituitarism	10 (2.3)
Single Axis Hypofunction	90 (21.0)
Two Axis Hypofunction	25 (5.8)
Three Axis Hypofunction	1 (0.2)

a Excluding cases of elevated prolactin.

b Excluding cases of decreased prolactin.

### 3.3 Preoperative clinical symptoms and relevant examination results

From **Table 4**, it is evident that among the 155 patients with preoperative symptoms of menstrual disorders, galactorrhea, or sexual dysfunction, only 40.6% had elevated serum prolactin levels. The majority (53.5%) had normal serum prolactin levels, with a mean of 65.33 ng/ml (range: 0.4-2125, standard deviation 238.83). To mitigate the influence of outliers, a truncated mean of 28.26 ng/ml was calculated to represent the central tendency of preoperative serum prolactin levels. Among these 155 patients, a lower proportion (14.2%) exhibited deficiency in the pituitary-gonadal axis function, with most (68.4%) displaying normal function. Additionally, 20 cases (12.9%) of elevated pituitary-gonadal axis function were associated with these symptoms. Among the 9 cases with preoperative Cushing’s syndrome, only 22.2% had elevated serum cortisol levels, with 77.8% exhibiting normal serum cortisol levels. The mean serum cortisol level was 208.29 ng/ml (range: 79.6-315.0, standard deviation 72.92). However, among the 75 patients with acromegaly, 86.7% had elevated growth hormone levels.

**Table 4:** Comparison of preoperative clinical symptoms and examination results in patients.

Clinical Features	Number of Cases (%)
Menstrual Disorders/Galactorrhea/Sexual Dysfunction(n=155)	
Hyperprolactinemia	63 (40.6)
Normal Serum Prolactin	83 (53.5)
Hypoprolactinemia	7 (4.5)
Mean Serum Prolactin Level	28.26
Immunohistochemistry PRL Positive	55 (35.5)
Gonadal Axis Hypofunction	22 (14.2)
Normal Gonadal Axis Function	106 (68.4)
Elevated Gonadal Axis Function	20 (12.9)
Cushing’s Syndrome (n=9)	
Hypercortisolism	2 (22.2)
Normal Serum Cortisol	7 (77.8)
Mean Serum Cortisol Level	208.29
Immunohistochemistry ACTH Positive	4 (44.4)
Acromegaly (n=75)	
Elevated Growth Hormone	65 (86.7)
Normal Growth Hormone	10 (13.3)
Immunohistochemistry GH Positive	48 (64.0)

### 3.4 Analysis of preoperative pituitary function factors

Among the cases mentioned earlier, it is evident that while 101 cases (23.6%) exhibited normal preoperative pituitary hormone levels, the remaining cases displayed various endocrine disturbances. Out of the total cases, 126 (29.6%) had deficiencies in at least one pituitary axis (excluding hypoprolactinemia), while the rest had normal function. Results from **Tables 5**, **6** reveal a gender-related association with preoperative endocrine disturbances (p < 0.05), with a higher prevalence in females (81.6%) compared to males (72.0%). Factors such as age, previous surgical history, presence of hypertension and diabetes, tumor size, tumor pathology, and tumor-related hemorrhage did not show significant relationships. **Tables 7** and **8** indicate that preoperative pituitary dysfunction (any axis) is related to gender, tumor size, and age. Female patients, those with larger tumor volumes, and older individuals were more prone to pituitary dysfunction. Additionally, there seems to be a potential link between a history of prior hospitalization (p = 0.053) and pituitary dysfunction occurrence.

**Table 5:** Impact factors of preoperative endocrine dysfunction: categorical variables.

Factor		Rate of Dysfunction	p-value
Gender	Male	72.0%	0.019
	Female	81.6%	
Comorbid Hypertension	Yes	73.4%	0.438
	No	77.2%	
Comorbid Diabetes	Yes	75.0%	0.826
	No	76.5%	
Times of Previous Surgery	0	77.4%	0.207
	1	67.3%	
	2	87.5%	
Tumor Size	Microadenoma	69.6%	0.393
	Macroadenoma	75.0%	
	Giant Adenoma	79.9%	
Pathological Classification	Non-functioning	71.2%	0.093
	Functioning	78.9%	
Tumor Apoplexy	Present	84.6%	0.205
	Absent	75.6%	

**Table 6:** Impact factors of preoperative endocrine dysfunction: numerical variables.

Factor	Normal	Dysfunction Rate	p-value
Age (year-old)	47.37	45.46	0.065
Tumor size(mm)	26.57	28.22	0.293

**Table 7:** Impact factors of preoperative endocrine hypofunction: categorical variables.

Factor		Hypofunction Rate	p-value
Gender	Male	25.0%	0.028
	Female	34.7%	
Comorbid Hypertension	Yes	35.1%	0.172
	No	27.8%	
Comorbid Diabetes	Yes	27.5%	0.777
	No	29.6%	
Previous Surgeries	Yes	40.0%	0.053
	No	27.7%	
Tumor Size	Microadenoma	8.7%	<0.001
	Macroadenoma	25.0%	
	Giant Adenoma	40.3%	
Pathological Classification	Non-functioning	33.8%	0.734
	Functioning	27.3%	
Tumor Apoplexy	Present	33.3%	0.576
	Absent	29.0%	

**Table 8:** Impact factors of preoperative endocrine hypofunction: numerical variables.

Factor	Hypofunction Rate	Normal	p-value
Age (year-old)	48.74	44.73	0.001
Tumor size(mm)	31.06	26.49	<0.001

### 3.5 Surgery and general postoperative conditions

**Table 9** provides an overview of the fundamental surgical and postoperative conditions. Surgical techniques employed included endoscopic endonasal tumor resection in 364 cases (85.0%) and craniotomy tumor resection in 64 cases (15.0%). The average operation duration was 62.20 minutes (range: 20-275, standard deviation 26.31) for endoscopic endonasal approaches and 256.20 minutes (range: 140-535, standard deviation 90.95) for craniotomy approaches. Complete resection was achieved in 31.8% of cases (136 cases), near-total resection in 65.7% (281 cases), and partial resection in 11 cases (2.6%). For endoscopic endonasal surgeries, 30 cases (8.2%) experienced sellar floor damage or cerebrospinal fluid leakage, and 21 cases (4.9%) required intraoperative blood transfusion. Postoperatively, 8 cases (1.9%) encountered surgical site bleeding, with 75% (6 cases) occurring after endoscopic endonasal procedures. Hematoma evacuation was performed in six cases, while conservative management was chosen for 2 cases, all leading to improved conditions.

**Table 9:** Surgical characteristics of 428 patients undergoing surgical treatment.

Item (n=428)	Number of Cases (%)
Surgical Approach	
Transnasal Sphenoid	364 (85.0)
Craniotomy	64 (15.0)
Surgical Duration (minutes)	
≤60	221 (51.6)
60-120	137 (32.0)
>120	70 (16.4)
Average Duration for Transnasal Sphenoid	62.20 minutes
Average Duration for Craniotomy	256.20 minutes
Tumor Resection	
Gross Total Resection	136 (31.8)
Near Total Resection	281 (65.7)
Partial Resection	11 (2.6)
Intraoperative Cerebrospinal Fluid Leakage (Transnasal Sphenoid, n=364)	30 (8.2)
Intraoperative Blood Transfusion	21 (4.9)
Postoperative Site Hemorrhagea ^a^	8 (1.9)

a Including 6 cases of transcranial surgery and 2 cases of craniotomy.

### 3.6 Postoperative changes in pituitary endocrine function

As depicted in **Table 10**, among the 420 cases with hormone retests within 7 days postoperatively, 59 cases (46.8%) of the 126 patients with preoperative deficiency in at least one endocrine axis experienced recovery of at least one axis. Among the 144 patients with preoperative elevation of at least one pituitary axis hormone, 92 cases (63.9%) achieved hormone relief. Among the 410 patients with no deficiency in any preoperative endocrine axis, 127 cases (31.0%) developed new-onset deficiency in at least one axis.

**Table 10:** Postoperative hormonal changes in cases reexamined within 7 days after surgery.

Category (n=420)	Number of Cases (%)
Restoration of Any Axis Function (n=126)	59 (46.8)
Pituitary-Adrenal Axis (n=71)	40^a^ (56.3)
Pituitary-Thyroid Axis (n=40)	19 (47.5)
Pituitary Growth Hormone Secretion (n=10)	8 (80.0)
Pituitary-Gonadal Axis (n=70)	7 (10.0)
Remission of Any Axis Function (n=144)	92 (63.9)
Pituitary-Adrenal Axis (n=6)	4 (66.7)
Pituitary-Thyroid Axis (n=5)	1 (20.0)
Pituitary Growth Hormone Secretion (n=71)	51 (71.8)
Pituitary-Gonadal Axis (n=67)	37 (55.2)
New Onset of Hypofunction in Any Axis (n=410)	127 (31.0)
Pituitary-Adrenal Axis (n=343)	109 (31.8)
Pituitary-Thyroid Axis (n=369)	10 (2.7)
Pituitary Growth Hormone Secretion (n=339)	2 (0.6)
Pituitary-Gonadal Axis (n=267)	20 (7.5)
Remission of Serum Prolactin^b^(n=137)	106 (77.4)
Preoperative PRL Mean: 18.79 ng/ml	
Postoperative PRL Mean: 7.56 ng/ml	P<0.001

a Includes cases transitioning from hypofunction to normal or hypofunction to hyperfunction, and vice versa.

b “Any Axis” mentioned above excludes prolactin.

In terms of individual endocrine axes, the highest recovery rate was observed for pituitary growth hormone secretion, with 8 out of 10 cases of preoperative growth hormone deficiency showing postoperative recovery. The next highest relief rate was seen for the pituitary-adrenal axis (56.3%) and pituitary-thyroid axis (47.5%). Prolactin exhibited the highest hormone relief rate (77.4%), followed by pituitary growth hormone (71.8%) and cortisol. Among the 6 cases with preoperative hypercortisolism, 4 cases achieved normalization of cortisol levels postoperatively. The highest rate of new-onset postoperative deficiency occurred in the pituitary-adrenal axis (31.8%), while the occurrence rates for other axes were relatively lower.

### 3.7 Factors influencing postoperative hormone relief

#### 3.7.1 Growth hormone relief

In the univariate analysis (**Tables 11**, **12**), postoperative relief of pituitary growth hormone was linked to tumor size, extent of surgical resection, age, and preoperative GH values (P < 0.05). However, it did not correlate with gender, history of prior hospitalization, tumor type, tumor-related hemorrhage, surgical approach, or operation duration. Smaller tumor diameters, greater rates of tumor resection, older age, and lower preoperative growth hormone values were associated with higher relief rates. Moreover, elevated preoperative FT4, FSH, and LH hormone levels, along with reduced preoperative PRL levels, were indicative of enhanced postoperative growth hormone relief rates (P < 0.05). Multivariate logistic regression analysis (**Table 13**) confirmed that postoperative growth hormone relief correlated with the extent of surgical resection and preoperative GH levels.

**Table 11:** Predictive factors of hormonal remission after surgery: categorical variables.

Category		Growth Hormone (n=71)		Prolactin (n=137)	
Feature	Group	Remission Rate (%)	p-value	Remission Rate (%)	p-value
Gender	Male	68.4	0.493	78.5	0.923
	Female	75.8		77.8	
Previous Surgeries	Yes	42.9	0.073	72.7	0.705
	No	75.0		78.6	
Pathological Classification	Non-functioning	54.5	0.272	84.1	0.244
	Functioning	75.0		75.3	
Tumor Apoplexy	Yes	0/1	1	57.9	0.034
	No	72.9		81.4	
Surgical Approach	Transnasal Sphenoid	74.2	0.132	78.6	0.771
	Craniotomy	40.0		75.0	
Tumor Resection	Gross Total Resection	87.5	0.023	75.5	0.744
	Near Total Resection	65.2		79.5	
	Partial Resection	0/1		1/1	

**Table 12:** Predictive factors of hormonal remission after surgery: numerical variables.

Clinical Feature		Growth Hormone		Prolactin	
Feature	Postoperative Status	Mean/Median	p-value	Mean/Median	p-value
Age	Remission	42.45	0.033	43.45	0.043
	No Remission	35.55		38.43	
Preoperative FT4	Remission	12.957	0.048	11.568	0.373
	No Remission	11.427		11.110	
Preoperative GH	Remission	17.558	<0.001	3.469	0.751
	No Remission	31.640		2.289	
Preoperative FSH	Remission	6.600	0.014	7.843	0.299
	No Remission	3,650		6.824	
Preoperative LH	Remission	4.602	0.010	2.608	0.876
	No Remission	1.687		2.600	
Preoperative PRL	Remission	15.897	0.018	38.877	<0.001
	No Remission	30.002		195.807	
Largest Tumor Diameter	Remission	18.33	<0.001	28.22	0.650
	No Remission	30.80		27.20	
Surgical Duration	Remission	59.58	0.061	77.73	0.346
	No Remission	90.39		90.26	

**Table 13:** Predictive factors of hormonal remission after surgery: multivariate logistic regression.

Category	Growth Hormone		Prolactin	
Feature	B Value	p-value	B Value	p-value
Age	-0.037	0.399	0.032	0.087
Largest Tumor Diameter	-0.069	0.100		
Preoperative GH	-0.095	0.017		
Preoperative PRL	0.001	0.894	-0.001	0.037
Preoperative FT4	-0.092	0.496		
Preoperative FSH	-0.028	0.776		
Preoperative LH	0.271	0.330		
Tumor Resection	-2.226	0.028		
Tumor Apoplexy			-0.791	0.166

#### 3.7.2 Prolactin relief

Analysis from **Tables 11** and **12** revealed that, in the univariate analysis, postoperative prolactin relief was linked to tumor-related hemorrhage, age, and preoperative PRL levels, while showing no connection to tumor pathology, size, or extent of surgical resection. The absence of tumor-related hemorrhage, advanced age, and lower preoperative PRL hormone levels suggested a higher postoperative prolactin relief rate. **Table 13** demonstrates that, in the multivariate analysis, postoperative prolactin relief was solely associated with preoperative PRL hormone levels.

### 3.8 Factors affecting postoperative functional recovery

#### 3.8.1 Adrenal axis

Univariate analysis (**Supplementary Tables 1**, **2**) indicated that postoperative recovery of pituitary-adrenal axis function had no correlation with tumor pathology, tumor size, extent of surgical resection, or preoperative hormone levels.

#### 3.8.2 Thyroid axis

Univariate analysis of predictive factors for recovery of pituitary-thyroid axis function (**Supplementary Tables 1**, **2**) demonstrated an association with preoperative FT4 levels and the number of deficient axes. Tumor size, pathology, and surgical variables had no impact on recovery rate. Higher preoperative FT4 levels and fewer preoperative deficient axes were linked to easier recovery of pituitary-thyroid axis function. Logistic regression analysis of these factors yielded a significance level of P = 0.004 < 0.05 for preoperative FT4, with B = 2.842, indicating consistent significance. However, the number of preoperative deficient axes had a P value of 0.070 > 0.05, suggesting no effect on recovery of pituitary-thyroid axis function.

### 3.9 Factors influencing new-onset postoperative deficiency

#### 3.9.1 Adrenal axis

In the univariate analysis (**Supplementary Tables 3**, **4**), the development of new-onset pituitary-adrenal axis deficiency postoperatively was influenced by a history of prior hospitalization and the interval between hormone retest and surgery. Patients with a history of previous surgery were more prone to experiencing new-onset pituitary-adrenal axis deficiency after surgery. Additionally, an extended interval between hormone retest and surgery was associated with a higher incidence of new-onset pituitary-adrenal axis deficiency within 7 days postoperatively. Multivariate analysis affirmed the significance of both factors with P values < 0.001, consistent with univariate analysis. The occurrence of new-onset pituitary-adrenal axis deficiency postoperatively was not tied to tumor size, type, or extent of surgical resection.

#### 3.9.2 Thyroid axis

Univariate analysis regarding new-onset pituitary-thyroid axis deficiency after surgery (**Supplementary Tables 3**, **4**) indicated a sole dependence on the preoperative FT4 level. Lower preoperative FT4 levels were associated with a higher likelihood of new-onset deficiency in the pituitary-thyroid axis postoperatively, without any connection to tumor size, surgical approach, or extent of surgical resection.

## 4 Discussion

### 4.1 Baseline characteristics

With the continuous advancement of medical technology and improvements in surgical skills, the effectiveness of surgical treatment for pituitary adenomas has significantly improved, leading to reduced complications (23–25). Mortality and disability rates have reached extremely low levels. Consequently, the evaluation of surgical outcomes in patients undergoing tumor resection primarily centers on the enhancement of postoperative quality of life, which is closely linked to the restoration of normal pituitary endocrine function.

This study’s subjects encompassed pituitary adenoma cases treated at a single center, benefiting from consistent medical expertise over a relatively brief time span. Minimized influence of medical and surgical techniques on outcomes ensured robust data stability and comparability. Hence, the study offers a reasonably dependable representation of postoperative hormone effects resulting from microscopic and endoscopic surgical interventions, adhering to current medical standards.

### 4.2 Potential factors affecting preoperative endocrine

Status The presence of preoperative endocrine disorders in pituitary adenoma patients exhibited a connection with gender (P < 0.05), attributing a higher likelihood to females. No substantial associations emerged with age, prior surgery history, hypertension, diabetes, underlying conditions, tumor dimensions, pathology, or tumor-related hemorrhage. This suggests that preoperative endocrine dysfunction in pituitary adenoma patients might bear a certain degree of randomness, not exclusively governed by tumor characteristics or disease progression.

Incidence of deficiency in any pituitary function axis prior to surgery correlated with gender, tumor size, and age. Male patients, those with larger tumor volumes, and older individuals faced a heightened risk of pituitary dysfunction. The frequency of pituitary target organ axis dysfunction was linked to tumor size, indicating that larger tumors exert more pressure on the normal pituitary, thus impeding its function. Furthermore, since the sample excluded children, older patients displayed more pronounced degradation of normal pituitary function due to age and heightened susceptibility to tumor-associated functional deficits.

The correlation between acromegaly and elevated growth hormone levels was remarkably strong, whereas patients with Cushing’s syndrome didn’t universally exhibit cortisol levels exceeding the upper normal range. Nevertheless, their levels remained relatively elevated within the range, aligning with clinical observations. The clinical manifestations of patients experiencing preoperative menstrual disorders, galactorrhea, or sexual dysfunction weren’t entirely elucidated by preoperative gonadal function and serum prolactin levels. However, the average serum prolactin level stood at 28.26 ng/ml, surpassing the upper normal limit, underscoring a close link between these symptoms and elevated serum prolactin levels, consistent with clinical observations.

### 4.3 Postoperative hormonal changes and influential factors

Examining postoperative hormone level shifts in pituitary adenoma patients—owing to their significant physiological impact and potential to affect quality of life—primarily centered on serum prolactin, growth hormone, cortisol relief, adrenal cortical function recovery, thyroid function recovery, gonadal function recovery, and incidence of new-onset cortisol and thyroid deficiency following surgery.

Our data demonstrates relatively high relief rates for serum prolactin, growth hormone, and cortisol after microscopic tumor resection (77.4%, 71.8%, and 66.7% respectively). Surgery significantly improved these endocrine imbalances, considerably impacting patients’ physical well-being. Gonadal axis hormone recovery after surgery proved less satisfactory, yet the prolactin relief rate at 77.4% offers sustained alleviation for symptoms like menstrual disorders and gonadal dysfunction. Prior studies indicated that factors affecting hormone relief post pituitary adenoma surgery encompassed age, preoperative hormone levels, tumor volume, tumor invasiveness, residual post-surgery tumor, and postoperative early-morning hormone levels (2, 5, 9, 10). Multifactorial analysis herein underscores that surgical resection rate (reflecting extent of tumor residue) and preoperative GH levels influence the recovery rate of growth hormone post-surgery. Single-factor analysis also suggests potential relevance of tumor size and age. For prolactin relief post-surgery, the pivotal factor remains preoperative PRL levels. Single-factor analysis hints at possible influence of tumor-related hemorrhage and patient age on relief rate. Limited data availability precluded an analysis of tumor invasiveness (26). These findings emphasize the significance of actively managing GH and PRL levels preoperatively, striving for maximal tumor resection to enhance relief rates for growth hormone and prolactin in postoperative pituitary adenoma patients.

Approximately half of patients with preoperative adrenal cortical dysfunction and thyroid dysfunction witnessed recovery (56.3% and 47.5% respectively). Most cases displayed varying hormone level elevation, notably enhancing patient quality of life. Earlier studies suggested recovery of pituitary target organ axis function in pituitary adenoma patients hinges on tumor size, patient age (27, 28), presence of intraoperative cerebrospinal fluid leakage, and tumor endocrine function (1). Yet, these studies didn’t differentiate recovery status of specific hormone secretions. We find that recovery of cortisol secretion function post-surgery isn’t significantly associated with patient age, tumor size, tumor function, surgical resection rate, or preoperative hormone levels. Advancements in diagnosis and treatment likely mitigate previous factors’ impact. Recovery of thyroid hormone secretion function post-surgery associates with higher preoperative FT4 levels, indicating enhanced likelihood of recovery for patients with less compromised function. Hence, patients with preoperative thyroid dysfunction are encouraged to seek treatment when tumor impact on pituitary-thyroid axis function is minimal.

Incidence rates of new-onset cortisol and thyroid hormone deficiency post-surgery weren’t negligible (31.3% and 2.7% respectively), necessitating short-term replacement therapy to sustain fundamental physiological needs and normal function after surgery. While some research suggested new-onset pituitary target organ axis dysfunction post-surgery was influenced by tumor volume (1), this study underscores no correlation between new-onset pituitary-adrenal axis or pituitary-thyroid axis dysfunction and tumor size. This likely reflects improved tumor resection techniques’ influence on outcomes. Data analysis underscores that new-onset cortisol secretion deficiency post-surgery significantly correlates with previous surgery history and interval between postoperative hormone reevaluation and surgery. This underscores the need for close monitoring and cortisol supplementation after surgery for reoperated patients. Notably, cortisol secretion deficiency onset generally transpires around the second day post-surgery, emphasizing the necessity of multiple cortisol reevaluations over several days.

## 5 Conclusion

Microsurgery demonstrates significant efficacy in ameliorating endocrine disturbances in patients afflicted with pituitary adenomas. Among the 428 cases examined in this study, postoperative remission rates for serum prolactin, growth hormone, and cortisol were notably elevated at 77.4%, 71.8%, and 66.7% respectively. Roughly half of the patients with preoperative adrenal insufficiency and thyroid dysfunction (56.3% and 47.5% respectively) exhibited recovery, with a majority achieving endocrine restoration. This confers a markedly positive influence on symptomatology and overall quality of life for individuals afflicted with pituitary adenomas.

The determinants impacting the postoperative remission rate of growth hormone encompass the surgical excision rate and preoperative GH levels. Moreover, the remission rate of prolactin subsequent to surgery correlates with preoperative PRL levels. Consequently, proactive management of GH and PRL levels preoperatively facilitates heightened rates of remission for growth hormone and prolactin post pituitary adenoma resection.

The recuperation of thyroid hormone secretion function post-surgery is closely associated with elevated preoperative FT4 levels. Encouragement is warranted for individuals with pituitary adenomas and preoperative thyroid dysfunction to proactively seek treatment when the tumor exerts a relatively minor impact on the pituitary-thyroid axis function.

Patients subjected to multiple surgeries manifest a heightened susceptibility to postoperative onset of cortisol secretion insufficiency. Therefore, a concerted effort towards comprehensive tumor resection and disease resolution in a singular surgical intervention is advised. Moreover, heightened vigilance should be directed towards postoperative monitoring and supplementation of cortisol for patients undergoing repeated or multiple surgical interventions.

## 6 Limitations and future prospects

Given the study’s focus on the perioperative period, longer follow-up is warranted to explore long-term effects. For cases where pituitary-gonadal axis function recovery proves suboptimal, a gender-based follow-up study, after excluding high prolactinemia’s influence, can uncover causal factors. Long-term follow-up for patients with new-onset pituitary-adrenal axis dysfunction post-surgery is essential to ascertain recovery potential, aiding comprehensive patient management and refining treatment strategies. Certain subgroups featured small sample sizes in this study, like cases with preoperative cortisol elevation (only six cases) and those with prior surgery history (only eight cases), constraining advanced statistical analysis and statistical power. For these low-incidence scenarios, accumulating more cases is vital to bolster analysis accuracy.

## Data availability statement

The raw data supporting the conclusions of this article will be made available by the authors, without undue reservation.

## Ethics statement

The studies involving humans were approved by the ethics committee of Peking University International Hospital. The studies were conducted in accordance with the local legislation and institutional requirements. Written informed consent for participation was not required from the participants or the participants’ legal guardians/next of kin in accordance with the national legislation and institutional requirements.

## Author contributions

MZ: Conceptualization, Formal Analysis, Software, Writing – original draft. KL: Data curation, Investigation, Writing – original draft. HN: Writing – review & editing. YZ: Resources, Writing – review & editing. CL: Methodology, Writing – review & editing.

## References

[1] Fatemi N Dusick JR Mattozo C McArthur DL Cohan P Boscardin J. Pituitary hormonal loss and recovery after transsphenoidal adenoma removal. Neurosurgery (2008) 63(4):709–18. doi: 10.1227/01.NEU.0000325725.77132.90 18981881

[2] Lonser RR Wind JJ Nieman LK Weil RJ DeVroom HL Oldfield EH. Outcome of surgical treatment of 200 children with Cushing's disease. J Clin Endocrinol Metab (2013) 98(3):892–901. doi: 10.1210/jc.2012-3604 23372173 PMC3590477

[3] Tamasauskas A Sinkunas K Bunevicius A Radziunas A Skiriute D Deltuva VP. Transsphenoidal surgery for microprolactinomas in women: results and prognosis. Acta Neurochir (Wien) (2012) 154(10):1889–93. doi: 10.1007/s00701-012-1450-x 22855071

[4] Ikeda H Watanabe K Tominaga T Yoshimoto T. Transsphenoidal microsurgical results of female patients with prolactinomas. Clin Neurol Neurosurg (2013) 115(9):1621–5. doi: 10.1016/j.clineuro.2013.02.016 23498159

[5] Primeau V Raftopoulos C Maiter D. Outcomes of transsphenoidal surgery in prolactinomas: improvement of hormonal control in dopamine agonist-resistant patients. Eur J Endocrinol (2012) 166(5):779–86. doi: 10.1530/EJE-11-1000 22301915

[6] Gondim JA Schops M de Almeida JPC de Albuquerque LAF Gomes E Ferraz T. Endoscopic endonasal transsphenoidal surgery: surgical results of 228 pituitary adenomas treated in a pituitary center. Pituitary (2010) 13(1):68–77. doi: 10.1007/s11102-009-0195-x 19697135

[7] Starke RM Raper DMS Payne SC Vance ML Oldfield EH Jane JA Jr. Endoscopic vs microsurgical transsphenoidal surgery for acromegaly: outcomes in a concurrent series of patients using modern criteria for remission. J Clin Endocrinol Metab (2013) 98(8):3190–8. doi: 10.1210/jc.2013-1036 23737543

[8] Sarkar S Jacob KS Pratheesh R Chacko AG. Transsphenoidal surgery for acromegaly: predicting remission with early postoperative growth hormone assays. Acta Neurochir (Wien) (2014) 156(7):1379–87. doi: 10.1007/s00701-014-2098-5 24781680

[9] Wang YY Higham C Kearney T Davis JRE Trainer P Gnanalingham KK. Acromegaly surgery in Manchester revisited–the impact of reducing surgeon numbers and the 2010 consensus guidelines for disease remission. Clin Endocrinol (Oxf) (2012) 76(3):399–406. doi: 10.1111/j.1365-2265.2011.04193.x 21824170

[10] Jane JJ Starke RM Elzoghby MA Reames DL Payne SC Thorner MO. Endoscopic transsphenoidal surgery for acromegaly: remission using modern criteria, complications, and predictors of outcome. J Clin Endocrinol Metab (2011) 96(9):2732–40. doi: 10.1210/jc.2011-0554 21715544

[11] Hofstetter CP Mannaa RH Mubita L Anand VK Kennedy JW Dehdashti AR. Endoscopic endonasal transsphenoidal surgery for growth hormone-secreting pituitary adenomas. Neurosurg Focus (2010) 29(4):E6. doi: 10.3171/2010.7.FOCUS10173 20887131

[12] Monteith SJ Starke RM Jane JA Jr Oldfield EH. Use of the histological pseudocapsule in surgery for Cushing disease: rapid postoperative cortisol decline predicting complete tumor resection. J Neurosurg (2012) 116(4):721–7. doi: 10.3171/2011.12.JNS11886 22283193

[13] Jagannathan J Smith R DeVroom HL Vortmeyer AO Stratakis CA Nieman LK. Outcome of using the histological pseudocapsule as a surgical capsule in Cushing disease. J Neurosurg (2009) 111(3):531–9. doi: 10.3171/2008.8.JNS08339 PMC2945523 19267526

[14] Fomekong E Maiter D Grandin C Raftopoulos C. Outcome of transsphenoidal surgery for Cushing's disease: a high remission rate in ACTH-secreting macroadenomas. Clin Neurol Neurosurg (2009) 111(5):442–9. doi: 10.1016/j.clineuro.2008.12.011 19200645

[15] Netea-Maier RT van Lindert EJ den Heijer M van der Eerden A Pieters GF Sweep CG. Transsphenoidal pituitary surgery viathe endoscopic technique: results in 35 consecutive patients with Cushing's disease. Eur J Endocrinol (2006) 154(5):675–84. doi: 10.1530/eje.1.02133 16645014

[16] Qu X Yang J Sun JD Mou CZ Wang GD Han T. Transsphenoidal pseudocapsule-based extracapsular resection for pituitary adenomas. Acta Neurochir (Wien) (2011) 153(4):799–806. doi: 10.1007/s00701-011-0961-1 21336808

[17] Qu X Wang M Wang G Han T Mou C Han L. Surgical outcomes and prognostic factors of transsphenoidal surgery for prolactinoma in men: a single-center experience with 87 consecutive cases. Eur J Endocrinol (2011) 164(4):499–504. doi: 10.1530/EJE-10-0961 21252173

[18] Schneider HJ Aimaretti G Kreitschmann-Andermahr I Stalla GK Ghigo E. Hypopituitarism. Lancet (2007) 369(9571):1461–70. doi: 10.1016/S0140-6736(07)60673-4 17467517

[19] Tornatore KM Logue G Venuto RC Davis PJ. Pharmacokinetics of methylprednisolone in elderly and young healthy males. J Am Geriatr Soc (1994) 42(10):1118–22. doi: 10.1111/j.1532-5415.1994.tb06219.x 7930339

[20] Lebrun-Vignes B Archer VC Diquet B Levron JC Chosidow O Puech AJ. Effect of itraconazole on the pharmacokinetics of prednisolone and methylprednisolone and cortisol secretion in healthy subjects. Br J Clin Pharmacol (2001) 51(5):443–50. doi: 10.1046/j.1365-2125.2001.01372.x PMC2014476 11422002

[21] Oruckaptan HH Senmevsim O Ozcan OE Ozgen T. Pituitary adenomas: results of 684 surgically treated patients and review of the literature. Surg Neurol (2000) 53(3):211–9. doi: 10.1016/S0090-3019(00)00171-3 10773251

[22] Esposito F Dusick JR Cohan P Moftakhar P McArthur D Wang C. Early morning cortisol levels as a predictor of remission after transsphenoidal surgery for Cushing’s disease. J Clin Endocrinol Metab (2006) 91(1):7–13. doi: 10.1210/jc.2005-1204 16234305

[23] Barker FN Klibanski A Swearingen B. Transsphenoidal surgery for pituitary tumors in the United States, 1996-2000: mortality, morbidity, and the effects of hospital and surgeon volume. J Clin Endocrinol Metab (2003) 88(10):4709–19. doi: 10.1210/jc.2003-030461 14557445

[24] Black PM Zervas NT Candia GL. Incidence and management of complications of transsphenoidal operation for pituitary adenomas. Neurosurgery (1987) 20(6):920–4. doi: 10.1227/00006123-198706000-00017 3614573

[25] Ciric I Ragin A Baumgartner C Pierce D. Complications of transsphenoidal surgery: results of a national survey, review of the literature, and personal experience. Neurosurgery (1997) 40(2):225–36. doi: 10.1097/00006123-199702000-00001 9007854

[26] Yuefei Z Feng F Gao D Liu W Fei Z He X. Clinical analysis of the resection of pituitary adenoma by bilateral nasal approach under neuroendoscope. Chin J Neurosurgical Dis Res (1995) 2013(03):250–3.

[27] Nomikos P Ladar C Fahlbusch R Buchfelder M. Impact of primary surgery on pituitary function in patients with non-functioning pituitary adenomas – a study on 721 patients. Acta Neurochir (Wien) (2004) 146(1):27–35. doi: 10.1007/s00701-003-0174-3 14740262

[28] Greenman Y Tordjman K Kisch E Razon N Ouaknine G Stern N. Relative sparing of anterior pituitary function in patients with growth hormone-secreting macroadenomas: comparison with nonfunctioning macroadenomas. J Clin Endocrinol Metab (1995) 80(5):1577–83. doi: 10.1210/jcem.80.5.7745003 7745003

