# Supplementary material for: Perioperative hormone level changes and their clinical implications in patients with pituitary adenoma: a retrospective study of 428 cases at a single center
Source: Front Endocrinol (Lausanne). 2023 Oct 30;14:1286020. doi: 10.3389/fendo.2023.1286020 (PMC10642766; doi:10.3389/fendo.2023.1286020)
Supplement: Supplementary file 1 [file Table_1.docx]

| **Supplementary Table 1. Predictive Factors of Postoperative Recovery in Pituitary Target Gland Axes: Categorical Variables** | | | | | |
| --- | --- | --- | --- | --- | --- |
| Clinical Feature | | Adrenal Axis (n=71) | | Thyroid Axis (n=40) | |
| **Feature** | **Group** | **Occurrence Rate (%)** | **p-value** | **Occurrence Rate (%)** | **p-value** |
| Gender | Male | 56.8 | 0.840 | 48.0 | 0.935 |
|  | Female | 59.3 |  | 46.7 |  |
| Previous Surgeries | Yes | 53.3 | 0.697 | 46.2 | 0.906 |
|  | No | 58.9 |  | 48.1 |  |
| Pathological Classification | Non-functioning | 61.5 | 0.623 | 52.9 | 0.554 |
|  | Functioning | 55.6 |  | 43.5 |  |
| Tumor Resection | Gross Total Resection | 65.0 | 0.564 | 75.0 | 0.281 |
|  | Near Total Resection | 56.3 |  | 41.4 |  |
|  | Partial Resection | 1/3 |  | 1/3 |  |

| **Supplementary Table 2. Predictive Factors of Postoperative Recovery in Pituitary Target Gland Axes: Numerical Variables** | | | | | |
| --- | --- | --- | --- | --- | --- |
| Clinical Feature | | Adrenal Axis | | Thyroid Axis | |
| **Feature** | **Postoperative Status** | **Mean/Median** | **p-value** | **Mean/Median** | **p-value** |
| Age | Remission | 45.22 | 0.376 | 44.84 | 0.076 |
|  | No Remission | 47.90 |  | 51.43 |  |
| Preoperative FT4 | Remission | 9.790 | 0.318 | 8.420 | ＜0.001 |
|  | No Remission | 10.392 |  | 7.087 |  |
| Largest Tumor Diameter | Remission | 31.80 | 0.928 | 33.21 | 0.183 |
|  | No Remission | 31.50 |  | 37.29 |  |
| Number of Hypofunctioning Axes | Remission | 1.76 | 0.322 | 1.79 | 0.047 |
|  | No Remission | 1.53 |  | 2.70 |  |

| **Supplementary Table 3. Predictive Factors of New-Onset Hypofunctioning Pituitary Target Gland Axes: Categorical Variables** | | | | | |
| --- | --- | --- | --- | --- | --- |
| Clinical Feature | | Adrenal Axis (n=346) | | Thyroid Axis (n=369) | |
| **Feature** | **Group** | **Occurrence Rate (%)** | **p-value** | **Occurrence Rate (%)** | **p-value** |
| Gender | Male | 31.7 | 0.975 | 2.6 | 1 |
|  | Female | 31.9 |  | 2.9 |  |
| Previous Surgeries | Yes | 55.6 | ＜0.001 | 4.3 | 0.360 |
|  | No | 28.2 |  | 2.5 |  |
| Pathological Classification | Non-functioning | 27.9 | 0.289 | 3.4 | 0.733 |
|  | Functioning | 33.6 |  | 2.4 |  |
| Tumor Apoplexy | Yes | 40.7 | 0.298 | 2.8 | 0.979 |
|  | No | 31.0 |  | 2.7 |  |
| Surgical Approach | Transnasal Sphenoid | 32.4 | 0.572 | 2.5 | 0.631 |
|  | Craniotomy | 28.6 |  | 4 |  |
| Tumor Resection | Gross Total Resection | 38.3 | 0.185 | 2.5 | 0.272 |
|  | Near Total Resection | 28.6 |  | 2.5 |  |
|  | Partial Resection | 28.6 |  | 1/8 |  |

| **Supplementary Table 4. Predictive Factors of New-Onset Hypofunctioning Pituitary Target Gland Axes: Numerical Variables** | | | | | |
| --- | --- | --- | --- | --- | --- |
| Clinical Feature | | Adrenal Axis | | Thyroid Axis | |
| **Feature** | **New-Onset** | **Mean/Median** | **p-value** | **Mean/Median** | **p-value** |
| Age | Hypofunction | 44.34 | 0.087 | 41.10 | 0.173 |
|  | No Hypofunction | 46.74 |  | 45.97 |  |
| Preoperative FT4 | Hypofunction | 12.391 | 0.526 | 10.890 | 0.018 |
|  | No Hypofunction | 12.512 |  | 12.339 |  |
| Preoperative COR | Hypofunction | 118.192 | 0.436 | 112.940 | 0.970 |
|  | No Hypofunction | 114.260 |  | 108.526 |  |
| Preoperative PRL | Hypofunction | 13.800 | 0.515 | 15.100 | 0.948 |
|  | No Hypofunction | 11.750 |  | 13.300 |  |
| Largest Tumor Diameter | Hypofunction | 27.85 | 0.415 | 30.80 | 0.214 |
|  | No Hypofunction | 26.83 |  | 26.91 |  |
| Surgery Duration | Hypofunction | 60.000 | 0.458 | 77.0 | 0.236 |
|  | No Hypofunction | 63.500 |  | 60.0 |  |
| Post-op Days for Follow-up | Hypofunction | 2.5 | ＜0.001 | 2.1 | 0.531 |
|  | No Hypofunction | 1.5 |  | 2.3 |  |
